# Awakening from Sleep with Numbness and Indescribable Odd Feeling: Nocturnal Panic Attack or Sleep-Related Epilepsy?

**DOI:** 10.3390/brainsci11020137

**Published:** 2021-01-21

**Authors:** Jae Young Park, Sangjoon Kang, Gyeong Seon Choi, Hayun Choi, Inha Hwang

**Affiliations:** 1 Veterans Health Service Medical Center, Department of Neurology, 53 Jinhwangdo-ro 61-gil, Gangdong-gu, Seoul 05368, Korea; funuri@hanmail.net (J.Y.P.); myidealis@naver.com (S.K.); 2 Department of Neurology, Bundang Jesaeng General Hospital, Seongnam, Gyeonggi 13590, Korea; nselis@naver.com; 3 Veterans Health Service Medical Center, Department of Psychiatry, 53 Jinhwangdo-ro 61-gil, Gangdong-gu, Seoul 05368, Korea; chhy0402@gmail.com

**Keywords:** nocturnal panic, sleep-related epilepsy, parasomnias

## Abstract

Paroxysmal events during sleep can be classified into parasomnias, sleep-related movements, psychiatric events, neurologic events, or medically related events. Diagnosis can be difficult because of the frequent overlap of clinical descriptors and lack of diurnal findings. We report a case of a 68-year-old man who presented to the hospital complaining of awakening from sleep with numbness, which was followed by an indescribable odd feeling. We discuss overlapping clinical features of nocturnal panic and sleep-related epilepsy.

## 1. Introduction

Paroxysmal events during sleep can be classified into parasomnias, sleep-related movements, psychiatric events, neurologic events, or medically related events [1]. These nocturnal events allow for a very broad differential diagnosis because of the frequent overlap of clinical descriptors and lack of diurnal findings [2]. These conditions should be carefully diagnosed among disorders with similar presentations but differences in pathophysiology, disease courses, and treatment. In particular, nocturnal panic disorder and auras related to epilepsy have significant subjective symptom overlap, making it difficult for clinicians to make an accurate diagnosis. Although psychiatric symptoms are well associated with patients diagnosed with epilepsy, there is no specific guideline on how to distinguish these. Furthermore, there are many cases in which abnormalities are not objectively identified via electroencephalography (EEG) or polysomnography (PSG), again making them difficult to differentiate. Panic attacks may, on rare occasions, be misdiagnosed by neurologists and treated as partial seizures, and partial seizures may be overlooked by psychiatrists and treated as a neurotic disorder [3]. We report a patient who presented to the hospital with repeated awakening from sleep with numbness in the extremities followed by an indescribable odd feeling.

## 2. Case

A 68-year-old man presented to our department of neurology with six months of repeated awakening from sleep due to numbness followed by a strange feeling. The event was described as an intolerable numbness in his upper and lower extremities. Following this sensory symptom, he felt an indescribable odd feeling. When expressing his symptoms, he used words such as “intolerable” and “feeling restless”. The event lasted about an hour. He had these stereotypical sensory and emotional symptoms every night in which the event occurred, but not during the daytime or before falling asleep. The event occurred about twice per week and lasted between thirty and sixty minutes each time. Events began one or two hours after sleep initiation and never occurred during the latter half of the patient’s sleep. The patient was able to recall the episodes clearly and denied vivid dreams during the events. His bedtime partner denied witnessing convulsive movements, talking or screaming during sleep, and unresponsiveness when an episode occurred; however, she witnessed his snoring three to four days per week. The patient reported that he has been a relatively good sleeper before these recent events. After the event occurred, he had anticipatory anxiety about sleep. He had a past medical history of unstable angina and hypertension. He had no previous history of seizure or psychiatric disorder and did not take any psychiatric medications. He did not drink alcohol or smoke (tobacco products). He had no family history of epilepsy, parasomnias, or psychiatric disorders. The patient reported that he had recently lost his job and was under significant stress. The score on the Beck Depression Inventory (BDI) was 22/63, which was indicative of moderate depression. Neurological examination showed as normal. His body mass index was 22.8 (kg/m^2^). Routine laboratory tests were normal except for mild anemia (hemoglobin 12.7 mg/dL). The serum ferritin level was normal (101.8 ng/mL). He first visited a cardiologist where he underwent transthoracic echocardiography and Holter monitoring, which were both normal.

**Figure 1:**
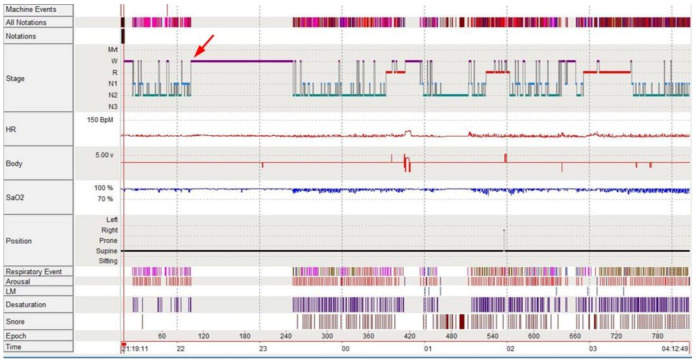
Hypnogram of the patient. The red arrow indicates the event of awakening from sleep with sensory and emotional symptoms, approximately 1 h after falling asleep.

**Figure 2:**
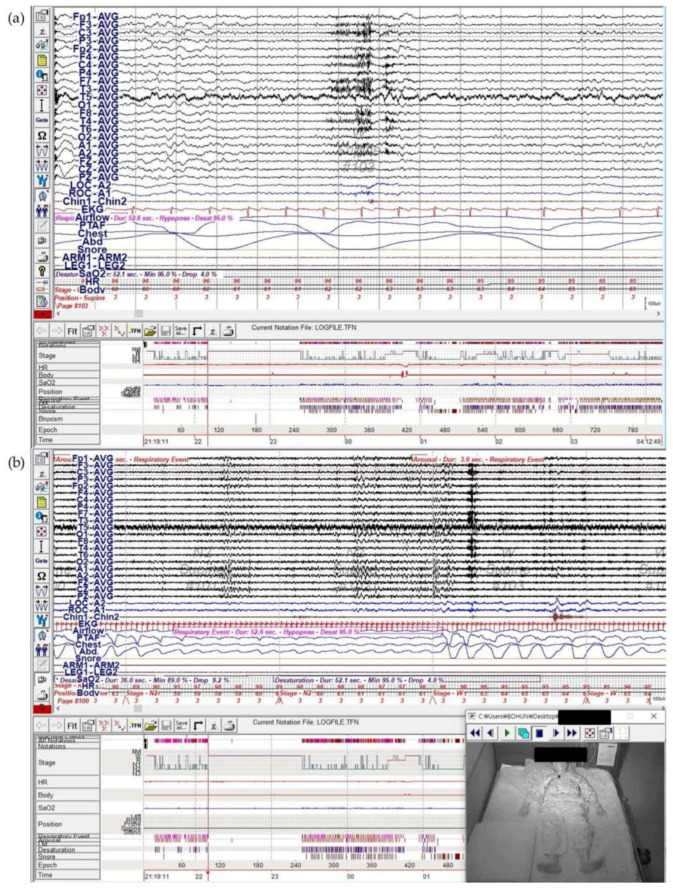
Nocturnal video PSG with extended EEG of the patient. Stereotypical recurrent events of awakening from sleep with numbness and indescribable odd feeling occurred in Epoch 103. (**a**) EEG channels in referential montage for 15 s, which reveals no epileptic discharge during the event. (**b**) PSG revealed obstructive hypopnea before the event. PSG: polysomnography, EEG: electroencephalography.

At our clinic, we performed overnight video PSG with extended EEG (EEG according to the international 10–20 system in referential montage) to differentiate between parasomnias, sleep-related movements, psychiatric events, sleep-related epilepsy, or other medically related events. On the night of the examination, the patient experienced the aforementioned sensory and emotional symptoms one hour after falling asleep, which aligned with awakening from stage N2 sleep (Figure 1 and Figure 2). The patient could not immediately fall back to sleep, and instead tossed and turned for an hour. There were no epileptiform discharges in EEG during the event, and the final overnight EEG was also normal. The patient’s heart rate showed slight elevation from 59 to 65 beats per min, which we did not think was significant. Abnormal sleep structure was observed, including frequent awakenings and low sleep efficiency (69.5%). PSG showed severe sleep apnea (apnea-hypopnea index of 49.4 per h). Brain magnetic resonance imaging (MRI) was normal, aside from mild small vessel disease.

Even though conflicting factors exist, making it difficult to distinguish epileptic attacks from panic attacks, considering the long duration of spells and recent history of stress, we concluded that the condition was of a functional nature rather than an epileptic symptom. We proceeded with continuous positive airway pressure (CPAP) treatment for sleep apnea, but the patient’s nocturnal paroxysmal events persisted. The frequency of the attacks decreased markedly only after administration of alprazolam 0.125 mg at bedtime. The patient was referred to the psychiatric department where he was treated with cognitive behavioral therapy (CBT) and selective serotonin reuptake inhibitor (SSRI), but had a follow-up loss after one session.

## 3. Discussion

Paroxysmal events during sleep can be classified into parasomnias, sleep-related movements, psychiatric events, neurologic events, or medically related events [1]. Diagnosis can be difficult because of the frequent overlap of clinical descriptors and the lack of diurnal findings. Patients may not follow the classic patterns of each disorder, and some patients may experience a mixture of events. Feature of spell such as time of night, length and frequency of occurrence and behavioral characteristics with each event, age of onset, past medical history, family history and specific laboratory data may help differentiate these disorders [1].

Our patient had relatively stereotypical recurrent attacks of sudden onset of awakening from early stages of sleep in the setting of numbness, an indescribable odd feeling, and preservation of consciousness. The patient’s symptoms, which were subjective, made the diagnosis difficult. First, our patient’s nocturnal events were possible in both panic attacks and seizures. It is known in general that both disorders can present with somatosensory, autonomic, or psychiatric symptoms [4]. Often, psychiatric symptoms such as anxiety, panic, and hallucinations are known to be a feature of the seizure itself, and auras during simple partial seizures include these psychiatric symptoms. Nocturnal panic attacks tend to be vividly recalled, to rarely recur more than once per night, and to have a prolonged duration [5]. Contrastingly, epileptic seizures occur in multiple spells during a single night, show shorter durations, and do not result in agoraphobia. Our patient fully recalled the experience of the event itself; the duration was relatively long (approximately an hour) and was improved by administration of an anxiolytic. These features were suggestive of nocturnal panic attacks rather than epileptic symptoms.

Nocturnal panic refers to awakening from sleep in a state of panic, where panic is defined as an abrupt and discrete period of intense fear or discomfort, accompanied by cognitive and physical symptoms of arousal [6]. Symptoms include: (1) palpitations, pounding heart, or accelerated heart rate; (2) sweating; (3) trembling or shaking; (4) sensations of shortness of breath or smothering; (5) feelings of choking; (6) chest pain or discomfort; (7) nausea or abdominal distress; (8) feeling dizzy, unsteady, light-headed, or faint; (9) chills or heat sensations; (10) paresthesias (numbness or tingling sensations); (11) derealization (feelings of unreality) or depersonalization (being detached from oneself); (12) fear of losing control or “going crazy”, and (13) fear of dying [7]. The symptom profile of nocturnal panic does not differ significantly from panic attacks that occur during wakeful states [8]. We found the following features in our patient that further supported the diagnosis of nocturnal panic attacks. Nocturnal panic is a non-rapid eye movement (NREM) event, usually occurring in late stage N2 or early stage N3 sleep [9]. Our patient’s event occurred at stage N2, which is consistent with nocturnal panic. Nocturnal panic is an abrupt awakening from sleep in a state of panic and without an obvious trigger, as in our patient’s case. Awakening from sleep followed by delayed-onset panic or nighttime arousals induced by environmental stimuli should not be considered as nocturnal panic [6]. Patients can recall their episodes of nocturnal panic, which frequently leads to distress and concern about recurrent attacks, as was seen in our patient [6].

Nakamura et al., (2013) studied whether nocturnal panic differs as a disease category from the coexistence of day panic and nocturnal panic attacks. They reported that patients who primarily experience panic attacks during the nocturnal sleep period belong to a mild and treatment-responsive subgroup of panic disorder, while patients with the coexistence of day panic and nocturnal panic attacks showed more severe panic disorder symptoms and worse treatment outcomes [10]. We think these different characteristics of nocturnal panic are consistent with our patient’s symptoms in that they were milder than typical day panic and were treatment-responsive to the anxiolytic.

Our patient’s nocturnal events could not be better explained by other sleep disorders such as night terror, REM sleep behavior disorder (RBD), or nightmares. Night terrors typically emerge within 2 h of sleep onset, similar to our patient’s event. However, they are usually limited to childhood, and the patient does not recall previous episodes of night terrors. RBD would be likely to occur towards the end of the sleep cycle, which was not the case in our patient’s recurring events. Sleep apnea is also an important disorder to be differentiated. Our patient had severe sleep apnea (AHI of 49.4). The possibility that the patient’s symptoms were attributable to sleep apnea was considered. However, since the patient’s recurrent sensory and emotional symptoms occurred after a stressor of losing a job recently, no improvement of his events after CPAP treatment, and improvement after anxiolytic administration, it was difficult to think that the events were caused by sleep apnea alone without nocturnal panic attacks. The limitation of the frequency of the patient’s nocturnal events to rarely more than one per night was also not consistent with the repetitive pattern of sleep apnea throughout the night [8].

It is essential to diagnose nocturnal panic so one can provide appropriate psychiatric measures to improve the quality of life [11]. Although we found an interviewer-administered nocturnal panic screen developed by Craske et al. (2005) to accurately diagnose nocturnal panic [6], research on the characteristics that distinguish it from other sleep-related events have been scarce until now. Therefore, further research is needed to better distinguish the clinical features of nocturnal panic for proper diagnosis and treatment.

## 4. Conclusions

The wide variety of paroxysmal events during sleep provides diagnostic challenges for physicians. For most patients, even though further investigation via PSG or video-EEG monitoring play an important role in identifying nocturnal events, it is essential for the clinician to perform an accurate history to guide diagnosis. Multidisciplinary approaches involving psychiatrists and neurologists, and to monitor response to treatment, may also be helpful. A possible direction to further this field of research is to develop a standard procedure for differentiating nocturnal panic from other sleep-related disorders.

## Data Availability

Not applicable.
